# Vertebral osteomyelitis as a hidden cause of persistent meningeal irritation in a patient with pneumococcal meningitis: A case report

**DOI:** 10.1097/MD.0000000000024705

**Published:** 2021-02-12

**Authors:** Mai Hamaguchi, Hiroaki Fujita, Keisuke Suzuki

**Affiliations:** Department of Neurology, Dokkyo Medical University, Japan.

**Keywords:** bacterial meningitis, infectious aortic aneurysm, *Streptococcus pneumoniae*, vertebral osteomyelitis

## Abstract

**Rationale:**

Pneumococcal meningitis generally develops from bacteremia and is often complicated by multiple organ infection.

**Patient concerns:**

A 62-year-old man with no previous medical history developed progressive disturbance of consciousness preceded by high-grade fever and headache for a few days.

**Diagnosis:**

The patient was diagnosed with pneumococcal meningitis based on meningeal irritation, polymorphonuclear cell-predominant pleocytosis of the cerebrospinal fluid (CSF) and a positive pneumococcal urinary antigen test at a different hospital. Despite the administration of meropenem and vancomycin, his consciousness worsened, and the patient was transferred to our hospital. Marked nuchal stiffness was noted. The patient showed a disturbance of consciousness, with a Glasgow Coma Scale score of E3V2M5. No significant cranial nerve palsy, motor weakness or sensory impairment was observed. CSF examination showed polynuclear cell-predominant pleocytosis of 755/μL. Transthoracic echocardiography revealed infectious endocarditis.

**Interventions:**

After the detection of penicillin-susceptible *Streptococcus pneumoniae*, the antibiotic regimen was changed to aminobenzylpenicillin 12 g/d and ceftriaxone 4 g/d, which improved the patient's consciousness and CSF findings. However, marked neck stiffness and neck pain persisted; we performed a systemic investigation that revealed cervical vertebral osteomyelitis and aortic aneurysm.

**Outcomes:**

After surgical treatment, the patient achieved complete remission of both conditions.

**Lessons:**

We should consider vertebral osteomyelitis as a potential complication of meningitis when nuchal stiffness persists despite an improvement in meningitis.

## 1 Introduction

Bacterial meningitis is a neurological emergency, with high rates of neurological sequelae and mortality, especially when treatment is delayed.^[1]^ Thus, bacterial meningitis requires systemic management, including appropriate antibiotic therapy in the early phase.^[1]^ It can be acquired spontaneously as a community-acquired bacterial meningitis in community or as a complication of invasive procedures or head trauma in the hospital (nosocomial bacterial meningitis). As patients with bacterial meningitis often show concomitant multiple organ infections, it should be treated as a systemic disease.^[1,2]^ We herein report a case of pneumococcal meningitis complicated by multiple organ infections. Notably, the systemic investigation revealed that complicated vertebral osteomyelitis was a hidden cause of sustained meningeal irritation in our patient.

## 2 Case report

A 62-year-old man who had not been vaccinated for *Streptococcus pneumoniae* developed high-grade fever and headache. He had no history of medical visits and was not on regular medication. Two days later, the patient was admitted to a hospital because of disturbance of consciousness. The patient was diagnosed with pneumococcal meningitis based on meningeal irritation, polymorphonuclear cell-predominant pleocytosis (92 cells/μL) with glucose levels of 0 mg/dL in the cerebrospinal fluid (CSF) and a positive pneumococcal urinary antigen test. Laboratory tests showed a white blood cell (WBC) count of 8580/μL (neutrophils, 93.4%) and C-reactive protein level of 30.12 mg/dL. According to the Japanese guidelines for bacterial meningitis,^[3]^ treatment with meropenem and vancomycin was initiated.

However, his consciousness worsened, and he was transferred to our hospital on the sixth day after onset. He was afebrile, and other vital signs were stable. His right wrist was swollen, and a cut on his right pollex pedis was found. Meningeal irritation including marked nuchal stiffness and Kernig's sign was observed. The patient showed a disturbance of consciousness, with a Glasgow Coma Scale of E3V2M5. No significant cranial nerve palsy, motor weakness or sensory impairment was observed. Tendon reflexes were bilaterally normal and negative for Babinski sign. Laboratory tests showed elevated inflammatory reactions: white blood cell count, 13,300/μL; C-reactive protein, 1.82 mg/dL; erythrocyte sedimentation rate, 21 mm (1 hour); and procalcitonin, 2.26 ng/mL. The D-dimer level was 10.6 μg/mL. Renal failure or diabetes mellitus was not present. The anti-HIV antibody test was negative. CSF examination showed polynuclear cell-predominant pleocytosis of 755/μL, a protein concentration of 976 mg/dL and a glucose level of 75 mg/dL (CSF/blood glucose, 0.56). The CSF was positive for pneumococcal antigen. An electrocardiogram showed sinus rhythm. Transthoracic echocardiography revealed infectious endocarditis (IE) in the aortic valve; however, no abnormalities were observed on chest computed tomography and brain magnetic resonance imaging (MRI) examinations on admission.

**Figure 1:**
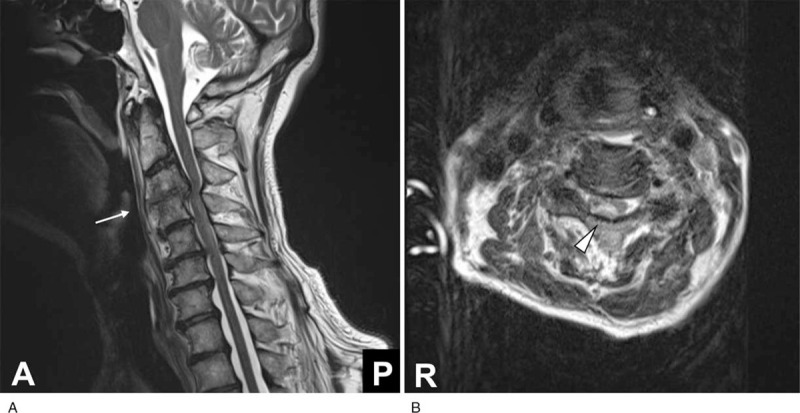
Magnetic resonance images of the cervical spine. In the T2-weighted sagittal image, vertebral osteomyelitis at the C3/4 level and compression of the spinal cord were detected (arrow) (A). The axial view of the slice shows myelomalacia at the compression site (arrowhead) (B).

Meropenem 6 g/d and vancomycin 2 g/d were continued, and dexamethasone 29.7 mg/d was added for 4 days. Penicillin-susceptible *Streptococcus pneumoniae* was detected in serum and CSF cultures, and according to antimicrobial susceptibility tests, we downescalated the antibiotic regimen to aminobenzylpenicillin 12 g/d and ceftriaxone 4 g/d. After 3 weeks of antibiotic therapy, his consciousness had improved; however, muscle weakness in the lower extremities and left-sided hearing impairment were noted. At 21 days after admission, his marked neck stiffness and neck pain persisted despite the improvement CSF findings, and Babinski sign became positive bilaterally. On cervical MRI, vertebral osteomyelitis at the C3/4 level was detected (Fig. 1). In addition, an infectious aortic arch aneurysm and Stanford B-type aortic dissection were noticed incidentally. Both vertebral osteomyelitis and infectious aortic aneurysm required surgical treatment. After the operations, his neck pain and meningeal signs disappeared. He was transferred to a rehabilitation hospital and returned to society 1 year after the onset of symptoms.

## 3 Discussion

We experienced a case of pneumococcal meningitis complicated by multiple organ infections. *Streptococcus pneumoniae* is the most common pathogen causing bacterial meningitis (60%) in adults despite the prevalence of pneumococcal vaccines.^[3,4]^ Pneumococcal meningitis shows poor prognosis, with a 20% to 37% mortality and 31% sequelae rate compared with other types of bacterial meningitis.^[5]^ Among the neurological sequelae, hearing impairment is the most common, followed by seizures, hydrocephalus, spastic/flaccid paralysis and other cranial nerve palsies.^[6]^ Weisfelt et al reported that the predictive factors of bacterial meningitis were as follows: older age, severe consciousness disturbance, tachycardia (>120 bpm), the presence of cranial nerve palsy, a CSF leukocyte count of less than 1000/μL and gram-positive cocci on CSF culture.^[7]^ Of those risk factors, our patient was of older age and had consciousness disturbance, cranial nerve palsy, a low CSF leukocyte count, and gram-positive cocci on CSF culture; the likelihood of an unfavorable outcome was approximately 80%.^[7]^

About 40% of pneumococcal IE cases are complicated by pneumococcal meningitis. ^[8]^ IE is a systemic infection that leads to the development of vegetation on the valves or endocardium and causes bacteremia, mycotic embolization and cardiac dysfunction. Delayed treatment can lead to multiple organ failure and unfavorable outcomes. *Streptococcus viridans*, *Staphylococcus aureus*, and *Enterococcus* spp. are the most common agents of IE, while *Streptococcus pneumoniae* accounts for less than 1%.^[8]^ Our patient with pneumococcal meningitis had multiple complications, such as vertebral osteomyelitis, aortic aneurysm and IE, presumably related to *Streptococcus pneumoniae* infection.

As his neck stiffness remained after the improvement of CSF findings, we suspected a comorbidity in the spine and performed a systemic investigation. Cervical computed tomography and MRI revealed vertebral osteomyelitis. Vertebral osteomyelitis, which can manifest as meningeal irritation signs and be complicated with meningitis, is a differential diagnosis of bacterial meningitis.^[9,10]^Table 1 shows the data of 13 reports with pneumococcal meningitis complicated by vertebral osteomyelitis. ^[11–21]^ Almost all patients were older, and the majority, including our patient, were not immune compromised. Complications in other organs included IE (n = 4), osteomyelitis of the wrist (n = 2), infectious aortic aneurysm (n = 2), and an iliopsoas/epidural abscess (n = 6). Three patients with severe vertebral destruction required spine surgery, and 2 patients with aortic aneurysm required vascular surgery. Regarding prognosis, the cases treated after the 1990  had good outcomes. The frequency of sequelae could not be assessed because of limited data.

**Table 1:** Patients with meningitis and vertebral osteomyelitis due to *Streptococcus pneumoniae*.

Patient number, ref	Patient (age, sex)	Underlying disease	Other symptoms/complications	Treatment	Outcome
^[11]^ Kaufman (1980)	58, NR	NR	IE	ABx, spine surgery	Died
^[12]^ Peterson (1987)	77, M	Fall injury	None	NFPC+GM→PC+CP, spine surgery	Died
^[13]^ Heard (1992)	61, F	None	IE	PCG→CLDM p.o.	Improved
^[14]^ Chemlal (1996)	52, M	Alcoholism, CHF	Acute bronchitis	CTX+RFP→CTRX+RFP→pristinamycin+RFP	Improved
^[15]^ Turner (1999)	79, F	None	IE	PCG+GM	Improved
^[16]^ Poyanli (2001)	51, F	None	None	CTRX, CT guided drainage	Improved
^[17]^ Brouwer (2008)	51, M	Depression	Arthritis of the left wrist	PC+DEX→flucloxacillin	Improved
^[17]^ Brouwer (2008)	39, F	Underwent operation of spondylosis	None	AMPC+DEX→flucloxacillin	Improved
^[18]^ Suzuki (2013)	69 (35-88), 9M/5F	diabetes mellitus, 35.7%; malignancy, 14.3%	epidural abscess, 14%; iliopsoas abscess, 21%; arthritis, 14%	β-lactam therapy	one patient (7.1%) died
^[19]^ Pires (2015)	61, M	HT	None	CTRX+VCM+ABPC	Improved
^[20]^ Gaini (2017)	66, F	Hypogammaglobulinemia due to MGUS	Pneumonia	CTRX+PCG+DEX→PCG, IVIg, Polysaccharide pneumococcal vaccination	Improved
^[21]^ Mitsutake (2018)	69, M	HT, MGUS, COPD, AAA	Right psoas abscess, the dilation of AAA	CTRX+VCM+ABPC+DEX, vascular surgery	Improved
Present case	62, M	None	IE, AAA, arthritis of the right wrist	MEPM+VCM+DEX→CTRX+ABPC, neck surgery, vascular surgery	Improved

AAA = abdominal aortic aneurysm, ABPC = ampicillin, ABx = antibiotics, AMPC = amoxicillin, CHF = congestive heart failure, CLDM = clindamycin, COPD = chronic obstructive pulmonary disease, CP = chloramphenicol, CTRX = ceftriaxone, CTX = cefotaxime, DEX = dexamethasone, F = female, GM = gentamicin, HT = hypertension, IE = infectious endocarditis, IVIg = intravenous immunoglobulin, M = male, MEPM = meropenem, MGUS = monoclonal gammopathy of undetermined significance, NFPC = nafcillin, NR = not reported, PC = penicillin, PCG = benzylpenicillin, RFP = rifampicin, VCM = vancomycin.

Vertebral osteomyelitis is mostly caused by hematogenous infection, and 5% of IE patients develop vertebral osteomyelitis.^[22]^ In vertebral osteomyelitis, fever and focal pain appear as the initial symptoms, and neurological symptoms typically emerge in the advanced phase.^[9]^ Its early diagnosis is difficult because in approximately half of patients, causative agents are not detected.^[23]^ In our patient, cervical vertebral osteomyelitis was detected at 21 days after admission. Despite proper medications, he developed cervical vertebral osteomyelitis accompanied by spondylotic myelopathy and required surgical treatment as in previous cases. Infectious aortic aneurysm was also detected incidentally and was considered a complication of pneumococcal IE. After vascular surgery followed by cervical anterior fusion, his neck pain and meningeal signs disappeared. He was transferred to a rehabilitation hospital and returned to society within a year.

## 4 Conclusion

Bacterial meningitis can cause critical complications, and it should be regarded as a systemic infectious disease. To avoid delays in diagnosing complications, we should consider vertebral osteomyelitis as a potential complication of meningitis when nuchal stiffness persists despite an improvement in meningitis signs, and urgent surgical treatment should be performed when necessary.

## Acknowledgments

We are grateful to Dr Yasushi Moridaira (Department of Orthopedics, Dokkyo Medical University) who performed the spine surgery and Dr Yusuke Takei (Department of Cardiovascular Surgery, Dokkyo Medical University) who performed the vascular surgery.

## Author contributions

**Conceptualization:** Mai Hamaguchi.

**Data curation:** Mai Hamaguchi.

**Formal analysis:** Hiroaki Fujita.

**Methodology:** Mai Hamaguchi, Hiroaki Fujita.

**Supervision:** Hiroaki Fujita, Keisuke Suzuki.

**Writing – original draft:** Mai Hamaguchi, Keisuke Suzuki.

**Writing – review & editing:** Hiroaki Fujita, Keisuke Suzuki.

## Abbreviations

Abbreviations: CSF = cerebrospinal fluid, IE = infectious endocarditis, WBC = white blood cell, CT = computed tomography, MRI = magnetic resonance imaging, PSSP = Penicillin-susceptible Streptococcus pneumoniae.

## References

[1] van de Beek D Brouwer M Hasbun R. Community-acquired bacterial meningitis. Nat Rev Dis Primers 2016;2:16074. 27808261 10.1038/nrdp.2016.74

[2] McGill F Heyderman RS Panagiotou S. Acute bacterial meningitis in adults. Lancet 2016;388:3036–47. 27265346 10.1016/S0140-6736(16)30654-7

[3] Ishikawa H Kamei S. Revised Japanese guidelines for the clinical management of bacterial meningitis. Clin Neurol 2014;54:1021–3. 10.5692/clinicalneurol.54.1021 25672697

[4] Oordt-Speets AM Bolijn R van Hoorn RC. Global etiology of bacterial meningitis: a systematic review and meta-analysis. PLoS One 2018;13:e0198772. 29889859 10.1371/journal.pone.0198772 PMC5995389

[5] van de Beek D de Gans J Spanjaard L. Clinical features and prognostic factors in adults with bacterial meningitis. N Engl J Med 2004;351:1849–59. 15509818 10.1056/NEJMoa040845

[6] Jit M. The risk of sequelae due to pneumococcal meningitis in high-income countries: a systematic review and meta-analysis. J Infect 2010;61:114–24. 20433866 10.1016/j.jinf.2010.04.008

[7] Weisfelt M van de Beek D Spanjaard L. A risk score for unfavorable outcome in adults with bacterial meningitis. Ann Neurol 2008;63:90–7. 17823938 10.1002/ana.21216

[8] de Egea V Muñoz P Valerio M. Characteristics and outcome of *Streptococcus pneumoniae* endocarditis in the XXI century: a systematic review of 111 cases (2000-2013). Medicine 2015;94:e1562. 26426629 10.1097/MD.0000000000001562 PMC4616835

[9] Miyazaki M Yoshiiwa T Kodera R. Clinical features of cervical pyogenic spondylitis and intraspinal abscess. J Spinal Disord Tech 2011;24:E57–61. 21909038 10.1097/BSD.0b013e318227ed9d

[10] Moritani T Kim J Capizzano AA. Pyogenic and non-pyogenic spinal infections: emphasis on diffusion-weighted imaging for the detection of abscesses and pus collections. Br J Radiol 2014;87:20140011. 24999081 10.1259/bjr.20140011 PMC4453136

[11] Kaufman DM Kaplan JG Litman N. Infectious agents in spinal epidural abscesses. Neurology 1980;30:844–50. 7191067 10.1212/wnl.30.8.844

[12] Peterson JA Paris P Williams AC. Acute epidural abscess. A J Emerg Med 1987;5:287–90. 10.1016/0735-6757(87)90352-4 2954559

[13] Heard SR Pickney J Tunstall-Pedoe DS. Pneumococcal endocarditis and disseminated infection. J Clin Pathol 1992;45:1034–5. 1452781 10.1136/jcp.45.11.1034 PMC495043

[14] Chemlal K Trouillet JL Carbon C. Vertebral osteomyelitis and meningitis due to a penicillin-resistant pneumococcal strain. Eur J Clin Microbiol Infect Dis 1996;15:893–5. 8997568 10.1007/BF01691227

[15] Turner DPJ Weston VC Ispahani P. *Streptococcus pneumoniae* spinal infection in Nottingham, United Kingdom: not a rare event. Clin Infect Dis 1999;28:873–81. 10825053 10.1086/515194

[16] Poyanli A Poyanli O Akan K. Pneumococcal vertebral osteomyelitis: a unique case with atypical clinical course. Spine 2001;26:2397–9. 11679828 10.1097/00007632-200111010-00020

[17] Brouwer MC de Gans J Heckenberg SGB. Vertebral osteomyelitis complicating pneumococcal meningitis. Neurology 2008;71:612–3. 18711116 10.1212/01.wnl.0000323929.02976.2b

[18] Suzuki H Shichi D Tokuda Y. Pneumococcal vertebral osteomyelitis at three teaching hospitals in Japan, 2003-2011: analysis of 14 cases and a review of the literature. BMC Infect Dis 2013;13:525. 24209735 10.1186/1471-2334-13-525 PMC3833677

[19] Pires A Kodra A Wisa D. A rare case of Pneumococcal Meningitis and Vertebral Osteomyelitis. Pract Neurol 2015;47:47–8.

[20] Gaini S Gudnason D Steig B. Meningitis, spondylodiscitis, pneumonia and septic shock with Streptococcus pneumoniae in a previously healthy woman with isolated IgG2-, IgG3-, IgA-deficiency and monoclonal gammopathy of undetermined significance. Infect Dis Rep 2018;10:7310. 29721239 10.4081/idr.2018.7310 PMC5907731

[21] Mitsutake A Matsumoto H Hatano K. Pneumococcal meningitis with vertebral osteomyelitis, iliopsoas abscess, and infected abdominal aortic aneurysm. Neurol Clin Neurosci 2018;6:23–5.

[22] Pigrau C Almirante B Flores X. Spontaneous pyogenic vertebral osteomyelitis and endocarditis: incidence, risk factors, and outcome. Am J Med 2005;118:1287. 10.1016/j.amjmed.2005.02.027 16271915

[23] Tali ET Oner AY Koc AM. Pyogenic spinal infections. Neuroimaging Clin N Am 2015;25:193–208. 25952173 10.1016/j.nic.2015.01.003

